# Supplementary material for: A Novel Mutation in the ADAMTS10 Associated with Weil–Marchesani Syndrome with a Unique Presentation of Developed Membranes Causing Severe Stenosis of the Supra Pulmonic, Supramitral, and Subaortic Areas in the Heart
Source: Int J Mol Sci. 2023 May 16;24(10):8864. doi: 10.3390/ijms24108864 (PMC10219133; doi:10.3390/ijms24108864)
Supplement: Supplementary file 1 [file ijms-24-08864-s001.zip › ijms-2375138-supplementary.pdf]

Supplementary Materials

Supplementary Table S1. Sixty shared homozygous variations with allele frequencies of less than 1% in the public databases (gnomad, ExAc browser, 1000 Genomes, NHLBI ESP Frequency, and dbSNP) between the sisters.

No.	Gene	Chr.	Position	Transcript Variant	Protein Variant
1	FNDC7	1	109255552	c.-4C>T	
2	IGSF3	1	117142869	c.1723T>C; c.1783T>C	p.W595R; p.W575R
3	F5	1	169519049	c.1601A>G; c.1601A>G *	p.Q534R; p.R534R
4	ARV1	1	231125863	n.314G>A; c.302G>A	p.G101E
5	LOC149373; TRIM67; AL109810.2	1	231323336	c.-23T>C; c.859-9781A>G; c.1045-9781A>G	
6	MCM6	2	136598443	c.2428T>C	p.Y810H
7	ZEB2	2	145162506	c.489C>T; c.417C>T; c.332-809C>T; c.486C>T	p.I139I; p.I163I; p.I162I
8	PDZRN3-AS1; PDZRN3	3	73674132	c.-156C>A; n.399-431G>T	
9	FRG2C	3	75714337	c.317T>G; c.314T>G	p.I105S; p.I106S
10	ZNF717; MIR4273	3	75786243	n.-1188C>T; c.277+4184G>A; c.2531G>A; c.256+4184G>A; c.2381G>A; c.2166+365G>A	p.C844Y; p.C794Y
11	ZNF717; MIR4273	3	75786354	c.2270C>T; n.-1077G>A; c.277+4073C>T; c.2166+254C>T; c.2420C>T; c.256+4073C>T	p.T757I; p.T807I

12	ZNF717	3	75787726	c.277+2701C>T; c.1048C>T; c.256+2701C>T; c.898C>T	p.R300C; p.R350C
13	ZNF717	3	75788010	c.277+2417T>G; c.256+2417T>G; c.614T>G; c.764T>G	p.V255G; p.V205G
14	ZNF717	3	75790513	c.170A>G; c.191A>G; c.41A>G	p.Y14C; p.Y57C; p.Y64C
15	ZNF717	3	75790822	c.-28T>C; c.123T>C; c.102T>C	p.D34D; p.D41D
16	ARL13B	3	93714763	c.59+15437C>T; c.105C>T; c.-63C>T; n.370C>T; n.374C>T; c.-179-7740C>T	p.T35T
17	MUC4	3	195508222	c.83-2896C>A; c.10229C>A; c.83-7046C>A	p.P3410H
18	MUC4	3	195508230	c.10219_10221delATG; c.83-7056_83-7054delATG; c.83-2906_83-2904delATG	p.M3407del
19	MUC4	3	195508237	c.83-2911A>C; c.83-7061A>C; c.10214A>C	p.D3405A
20	MUC4	3	195512227	c.83-11194_83-11051delTGGTGACACCACGCCGCTTCCTGTCACCGATACTTCCTCAGCATCCACAGGTCAGGACACCCCTCTTCCTGTCACCAGCCTTTCCTCAGTA TCCACAGGTGACACCACGCCTCTTCCTGTCAC	p.D2029_G2076del

				TAACCCTTCCT CAGCATCCAC; c.6081_6224delT GGTGACACCA CGCCGCTTCCT GTCACCGATAC TTCCTCAGCAT CCACAGGTCA GGACACCCCTC TTCCTGTCACC AGCCTTTCCTC AGTATCCACA GGTGACACCA CGCCTCTTCCT GTCACTAACCC TTCCTCAGCAT CCAC; c.83-7044_83-6901delTGGTGA CACCACGCCG CTTCCTGTCAC CGATACTTCCT CAGCATCCAC AGGTCAGGAC ACCCCTCTTCC TGTCACCAGCC TTTCCTCAGTA TCCACAGGTG ACACCACGCCT CTTCCTGTCAC TAACCCTTCCT CAGCATCCAC	
21	MUC4	3	195512302	c.83-7119_83-6976delGGTCAC CGACACTTCCT CAGTATCTACA GGACAGGCCA CCCCTCTTCCT GTCACCAGCCT TTCCTCAGCAT CCACTGGTGAC ACCACGCCGCT TCCTGTCACCG ATACTTCCTCA GCATCCACAG GTCAGGACAC CCCTCTTCC; c.6006_6149delG GTCACCGACA CTTCCTCAGTA	p.D2005_T2052del

				TCTACAGGAC<br>AGGCCACCCCT<br>CTTCCTGTCAC<br>CAGCCTTTCCT<br>CAGCATCCACT<br>GGTGACACCA<br>CGCCGCTTCCT<br>GTCACCGATAC<br>TTCCTCAGCAT<br>CCACAGGTCA<br>GGACACCCCTC<br>TTCC; c.83-<br>11269_83-<br>11126delGGTCA<br>CCGACACTTCC<br>TCAGTATCTAC<br>AGGACAGGCC<br>ACCCCTCTTCC<br>TGTCACCAGCC<br>TTTCCTCAGCA<br>TCCACTGGTGA<br>CACCACGCCG<br>CTTCCTGTCAC<br>CGATACTTCCT<br>CAGCATCCAC<br>AGGTCAGGAC<br>ACCCCTCTTCC	
22	MUC4	3	195514078	c.83-12902C>A;<br>c.83-8752C>A;<br>c.4373C>A	p.P1458H
23	ZNF732	4	265222	c.1424G>A	p.R475K
24	PCDHGA1	5	140710816	c.565C>T	p.P189S
25	SFT2D1	6	166743667	n.219C>T;<br>c.132C>T;<br>n.210C>T	p.G44G
26	NFE2L3	7	26192337	c.219C>G	p.H73Q
27	C9orf131	9	35043075	c.305T>C;<br>c.449T>C;<br>c.344T>C;<br>c.230T>C	p.I102T; p.I77T;<br>p.I115T; p.I150T
28	RGP1; GBA2	9	35748436	c.266G>C; c.-<br>989C>G	p.C89S
29	ADAMTSL2	9	136434598	c.2313A>G;<br>c.2640A>G	p.V880V;<br>p.V771V

30	LOC101448202; MIR3689C	9	137741153	n.39_62dupCGTGGTTCCTGGGAGGTGTGATAT; n.70+23218_70+23241dupCGTGGTTCCTGGGAGGTGTGATAT	
31	LIPA; IFIT3	10	91099466	c.898G>C; c.1054G>C; c.61+73082C>G	p.V352L; p.V300L
32	IFIT1; LIPA	10	91152372	c.-426T>A; c.-305T>A; c.-195T>A; c.61+20176A>T; c.-98T>A; c.-300T>A	
33	BAD	11	64037602	c.*79C>G	
34	KCNK7	11	65361256	c.409G>A	p.V137M
35	TAS2R30; PRH1-TAS2R14; PRH1; PRH1-PRR4	12	11286282	n.204+37739C>T; c.- 295+37739C>T; n.190+37739C>T; c.-134+37739C>T; c.562C>T	p.L188F
36	TAS2R30; PRH1-TAS2R14; PRH1; PRH1-PRR4	12	11286323	n.204+37698A>T; c.-134+37698A>T; c.521A>T; c.-295+37698A>T; n.190+37698A>T	p.H174L
37	ZNF26	12	133587980	c.1614C>T; c.1419C>T; c.1515C>T; c.1455C>T	p.T485T; p.T538T; p.T505T; p.T473T
38	SKA3; MRPL57	13	21751164	c.109C>T; c.-548G>A	p.R37C
39	RYR3	15	34080588	c.9759C>T	p.D3253D
40	GRIN2A	16	9892300	c.2190C>T	p.Y730Y
41	RMI2; TNP2	16	11362549	c.516+18914G>A; c.*160C>T; c.400+171C>T	
42	MKS1	17	56292123	c.464G>A; c.494G>A; c.-94-	p.R155H; p.R165H

				375G>A	
43	PPM1E	17	57046980	n.793C>G; c.864C>G	p.L288L
44	ACE	17	61561853	c.1872C>T; c.-376C>T	p.P624P
45	ZNF397; ZSCAN30	18	32833367	c.*47G>C; c.647-829C>G	
46	FHOD3	18	34298542	c.2705G>A; c.2756G>A; c.3281G>A	p.R919Q; p.R1094Q; p.R902Q
47	TPGS2	18	34378323	c.*62A>G; c.552+89A>G; c.657+89A>G; c.382+7014A>G; c.528+89A>G	
48	ADAMTS10	19	8670100	c.232T>C	p.Y78H
49	OR1M1	19	9204024	c.104_108delACCTG	p.Y35fs*34
50	TRPM4	19	49685865	c.949G>A; c.1294G>A; c.232G>A; c.772G>A; c.-260G>A	p.A432T; p.A317T; p.A78T; p.A258T
51	TRPM4	19	49691898	c.682G>A; c.136G>A; c.1399G>A; c.1744G>A; c.1222G>A	p.G46S; p.G408S; p.G228S; p.G467S; p.G582S
52	KIR3DL3	19	55239223	c.502G>A	p.V168I
53	KIR2DL3	19	55250036	c.26T>C	p.V9A
54	TTI1	20	36640902	c.1317C>T	p.D439D
55	KIAA1755	20	36842004	n.1615G>A; c.1996G>A; c.3043G>A	p.A1015T; p.A666T
56	PKIG	20	43246999	c.225G>A	p.S75S
57	SPO11	20	55906997	c.240C>T; c.132-1247C>T	p.N80N
58	POTED	21	14982952	c.403A>G	p.I135V

59	POTED	21	15013735	c.1603A>G	p.M535V
60	NLGN4Y	Y	16952347	c.1656T>C; n.2130T>C; c.1716T>C; c.1827T>C; n.1895T>C; c.1152T>C	p.N552N; p.N609N; p.N572N; p.N384N

Supplementary Table S2. Number of variants in the exome sequence of patients III-1 and III-2 with an allele frequency of less than 1% in the public databases: gnomAD browser, 1000 Genomes, ExAC, EVS, and the reason for their negation as the causative mutation.

The Cause for Negation	Number of Variants That Were Negated
Common variants, according to our internal laboratory Exome database of the Bedouin population	11
Common variants, according to a database of healthy Saudi individuals with LOF in various genes (Alsalem et al. 2013 (29))	8
A variation on chromosome Y (the patients are females)	1
Homozygote individual/s in the genomAD browser data	15
One or two sisters are heterozygote on IGV (instead of homozygotization for the variation)	12

Supplementary Table S3. Variations remaining after first filtration and reason for negation. In red – variations which were negated since they appeared on common variants databases (in their heterozygote variation) / the position of the variation was deep in the intron / the variation does not change the amino acid on the exon/family segregation - one or more healthy individuals were found homozygote for the mutated allele.

**Gene Symbol**	**Transcript Variant**	**MGI**	**Decision**
FNDC7	c.-4C>T	Homeostasis/metabolism nervous system	Appears in a heterozygote variation on a common variants database + insignificant position in the UTR
LOC149373	c.-23T>C	Behavior/neurological	Insignificant position in the intron
NFE2L3	c.219C>G	Normal phenotype	Appears in a heterozygote variation on a common variants database
TNP2	c.-516+18914G>A	TNP2 cellular reproductive system normal phenotype	Insignificant position in the intron
MKS1	c.464G>A	http://www.informatics.jax.org/humanDisease.shtml Meckel Syndrome, Type 1 OMIM:249000 https://hpo.jax.org/app/browse/disease/OMIM:249000	Extended family segregation, affected individuals III-3+III-4 are heterozygotes to the variation
PPM1E	c.864C>G p.L288L	No model	No change of amino acid on the exon
ACE	c.1872C>T p.P624P	Cardiovascular but not with skeletal problems	No change of amino acid on the exon
TPGS2	c.552+89A>G;	Behavior/neurological reproductive system skeleton- decreased bone mineral content	Insignificant position in the intron

ADAMTS10	c.232T>C p.Y78H	Aortic valve stenosis/Mitral regurgitation/Pulmonic stenosis/Abnormality of cardiovascular s/Ventricular septal defect/Patent ductus arteriosus<br>Weill–Marchesani syndrome https://medlineplus.gov/genetics/condition/ weill-marchesani-syndrome/ date 23 March 2023	Possible candidate further verification
OR1M1	c.104_108delACCTG p.Y35fs*34	No model	Possible candidate further verification
TTI1	c.1317C>T p.D439D	No model	No change of amino acid on the exon
KIAA1755	c.1996G>A	No model	Appears in a heterozygote variation on a common variants database
SPO11	c.240C>T	Cellular/ endocrine/exocrine glands/hematopoietic system/homeostasis/metabolism/immune system/mortality/aging/reproductive system	Appears in a heterozygote variation on a common variants database
